# Designing All Graphdiyne Materials as Graphene Derivatives: Topologically Driven Modulation of Electronic Properties

**DOI:** 10.1021/acs.jpcc.1c04238

**Published:** 2021-07-15

**Authors:** Patrick Serafini, Alberto Milani, Davide M. Proserpio, Carlo S. Casari

**Affiliations:** † Dipartimento di Energia, Politecnico di Milano, via Ponzio 34/3, 20133 Milano, Italy; ‡ Dipartimento di Chimica, Università degli Studi di Milano, 20133 Milano, Italy; § Samara Center for Theoretical Materials Science (SCTMS), Samara State Technical University, 443100 Samara, Russia

## Abstract

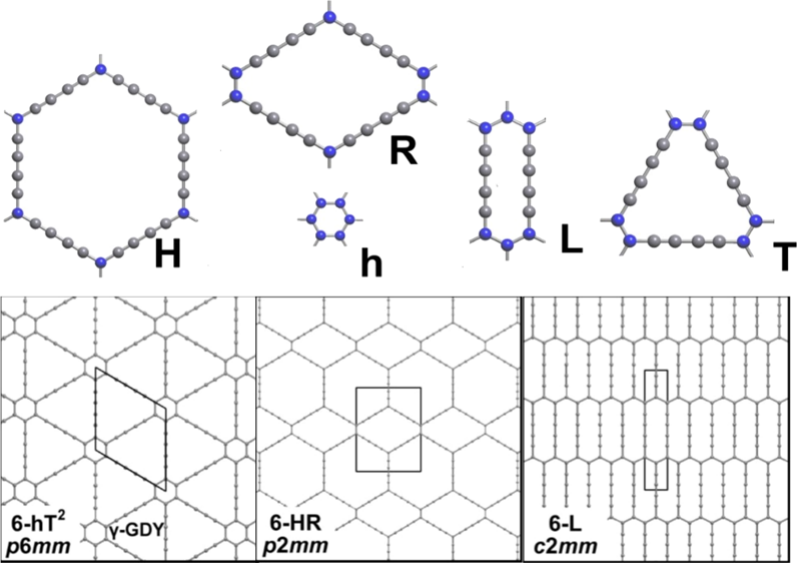

Designing new 2D systems with tunable properties is an important subject for science and technology. Starting from graphene, we developed an algorithm to systematically generate 2D carbon crystals belonging to the family of graphdiynes (GDYs) and having different structures and sp/sp^2^ carbon ratios. We analyze how structural and topological effects can tune the relative stability and the electronic behavior, to propose a rationale for the development of new systems with tailored properties. A total of 26 structures have been generated, including the already known polymorphs such as α-, β-, and γ-GDY. Periodic density functional theory calculations have been employed to optimize the 2D crystal structures and to compute the total energy, the band structure, and the density of states. Relative energies with respect to graphene have been found to increase when the values of the carbon sp/sp^2^ ratio increase, following however different trends based on the peculiar topologies present in the crystals. These topologies also influence the band structure, giving rise to semiconductors with a finite band gap, zero-gap semiconductors displaying Dirac cones, or metallic systems. The different trends allow identifying some topological effects as possible guidelines in the design of new 2D carbon materials beyond graphene.

## 1 Introduction

Carbon materials and their nanostructures played a relevant role in the science and technology of the last two decades: from fullerenes to carbon nanotubes and from polyconjugated polymers to graphene, the so-called “era of carbon allotropes” has been enlightened by groundbreaking results and Nobel prizes, paving the way to many interesting research topics.^1^ In the last years, the interest of many scientists has been directed toward exotic forms of carbon, including systems based on 1D sp-hybridized carbon (variously referred to as carbyne, carbon-atom wires, polyynes, cumulenes, ...) and on hybrid sp–sp^2^ carbon systems. These investigations focused both on the fundamental properties and the potential applications in different fields, showing promising perspectives for the near future.^2−6^ Graphyne (GY) and graphdiyne (GDY) represent 2D carbon crystals with sp–sp^2^ carbon atoms.^7−14^

They can be constructed as possible modification of graphene by interconnecting sp^2^-carbon hexagons with linear sp-carbon chains of different lengths (a single or a double acetylenic bond for GY and GDY, respectively), generating new systems with peculiar and tunable electronic and optical properties. Moreover, starting from GY and GDY, many other ideal 2D hybrid sp–sp^2^ carbon systems can be proposed by playing with geometry and topology, offering countless possibilities in the design and tailoring of carbon allotropes, both theoretically and experimentally.

Early theoretical studies on GY- and GDY-based systems were reported in 1987^15^ and recently with modern computational methods to shed light on their properties.^16−20^ These structures are a part of a larger family of two-dimensional π-conjugated covalent organic frameworks (COFs), showing the occurrence of Dirac cones,^21,22^ flat bands, and tunable bands gap. Such phenomena observed in the electronic structure of COFs have been explained based on peculiar topological effects also in connection to their influence on the charge transport behavior.^23−26^

Several papers report on the prediction of properties of γ-GDY mainly through density functional theory (DFT) calculations.^5,6,27−32^ From the experimental side, synthetic bottom-up approaches have been successfully employed to produce sub-fragments of GDY of different topologies and dimensions, in particular, by Haley and co-workers.^33−40^ Later, different papers reported the preparation and characterization of extended 2D GDY sheets prepared through organometallic synthesis techniques, showing promising routes to the production of these systems, even though significant efforts should still be put to further investigate and understand their properties.^41−43^ Recent advances in on-surface synthesis allow the preparation of hybrid sp–sp^2^ carbon nanostructures and their atomic-scale investigation with surface science techniques.^9,42,44−46^ The recent possibilities to realize new systems have opened new opportunities well beyond the sole investigation of their fundamental properties. Hence, the identification of possible guidelines to support the synthetic efforts is mandatory in a knowledge-based research approach. In addition, to develop a new class of 2D carbon materials, some relevant open issues still need to be addressed. First, how many different 2D crystal structures can be possible? What about their stability? Which structures are metallic, semimetallic with Dirac cones, or semiconducting? Is there any relation between the crystal structure and the electronic behavior?

To answer these questions, we have developed an algorithm to systematically generate new hybrid sp–sp^2^ carbon structures as modifications of graphene by introducing linear diacetylenic units. By DFT calculations on the geometries so generated, we performed geometry optimization, evaluation of the relative stability, and prediction of the electronic band structure, gap, and density of states (DOS). We analyzed a total of 26 structures, more than half not previously identified, and we outlined metallic, semimetallic with Dirac cones, and semiconducting systems grouped on the basis of topological features. The identification of new 2D carbon structures and the topology-based electronic properties give further insights into the design and understanding of new hybrid sp–sp^2^ carbon 2D materials.

## 2 Theoretical Details

To identify systematically all the sp–sp^2^ carbon systems in the GDY family, we used ToposPro^47^ to generate subnets of graphene where bonds are deleted in all possible ways. This procedure was used in the past to generate uninodal and binodal nets.^48,49^ Based on the 2D crystal structures selected by ToposPro, periodic boundary condition (PBC) DFT simulations have been carried out by employing CRYSTAL17^50,51^ to optimize the geometry (both the atomic position and cell parameters) and compute the electronic band structure and DOS. To this end, we adopted the PBE0 hybrid exchange–correlation functionals together with 6-31G(d) Gaussian basis sets.^52^ This level of theory has been chosen according to our previous investigations of the structural and vibrational properties of γ-GDY and related nanoribbons, where the results obtained using different functionals and basis sets have been compared.^31^ When using the 6-31G(d) basis set in PBC-DFT simulations with the CRYSTAL code, the exponent of the diffuse sp orbitals of carbon atoms has been increased from 0.1687144 to 0.187 bohr^–2^ to avoid convergence problems in the SCF, due to basis set linear dependencies.^53^ Considering the other simulation parameters, the tolerance on integral screening has been fixed to 9,9,9,9,80 (TOLINTEG parameters), while the shrink parameters defining Monkhorst–Pack and Gilat sampling points have been fixed to 100 and 200 for the calculation of the band structure and DOS, respectively. Depending on the crystalline structure (orthorhombic, monoclinic, and hexagonal), the main three paths and special points in the Brillouin zone were chosen. Band structures and DOS were plotted using the program CRYSPLOT, a visualization environment for plotting properties of crystalline solids as computed through the CRYSTAL code (http://crysplot.crystalsolutions.eu/).

Similar to ref (23), the data here reported have been obtained using the PBE0 functional, taking advantage of the improvement obtained by means of hybrid functionals in the description of ground-state electronic properties. Even if a further improvement could be obtained by employing the HSE06 functional,^54^ in our previous investigation on γ-GDY and its nanoribbons, we verified if both PBE0 and HSE06 are able to describe the same trends for band gaps, with a larger overestimation of PBE0 ones with respect to benchmark values computed by the GW method.^31^ For some peculiar structures, full geometry optimization and band structure and DOS calculations have been carried out also using the HSE06 functional and compared with PBE0 results.

## 3 Results and Discussion

### 3.1 Construction of GDY Crystals as Graphene Derivatives

**Figure 1:**
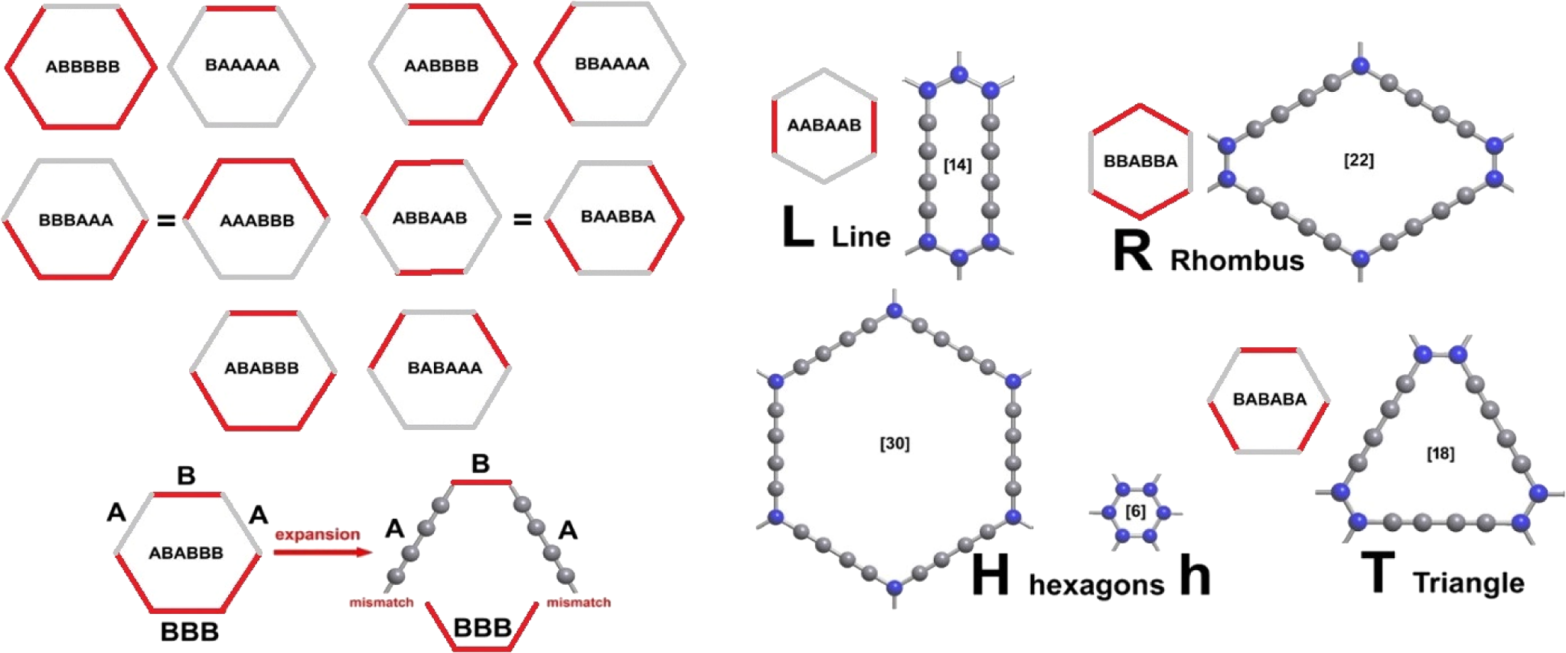
(Left) The eight distinct possible configurations that upon expansion give highly distorted hexagons that are discarded in our generation of GDY-like layers. A and B indicate non-equivalent sides of the hexagon units: B should be considered as a true CC bond, while A represents the diacetylene unit (C_4_) of four sp carbon atoms. The configurations are shown as “dual” couples, that is, reversing the role of A vs B. Two couples do not generate a new configuration, for example, BBBAAA = AAABBB. (Right) Four configurations that upon expansion gave undistorted six-sided polygons. The value in the square bracket is the total number of carbon atoms in the ring that uniquely define each building block, h = [6], H = [30]; L = [14]; T = [18], and R = [22]. Line (L) and rhombus (R) blocks are “dual”, so for each layer containing them, we can substitute each L with an R without significant distortions. The definition proposed here follows the one reported in the work by Park et al.^17^

**Figure 2:**
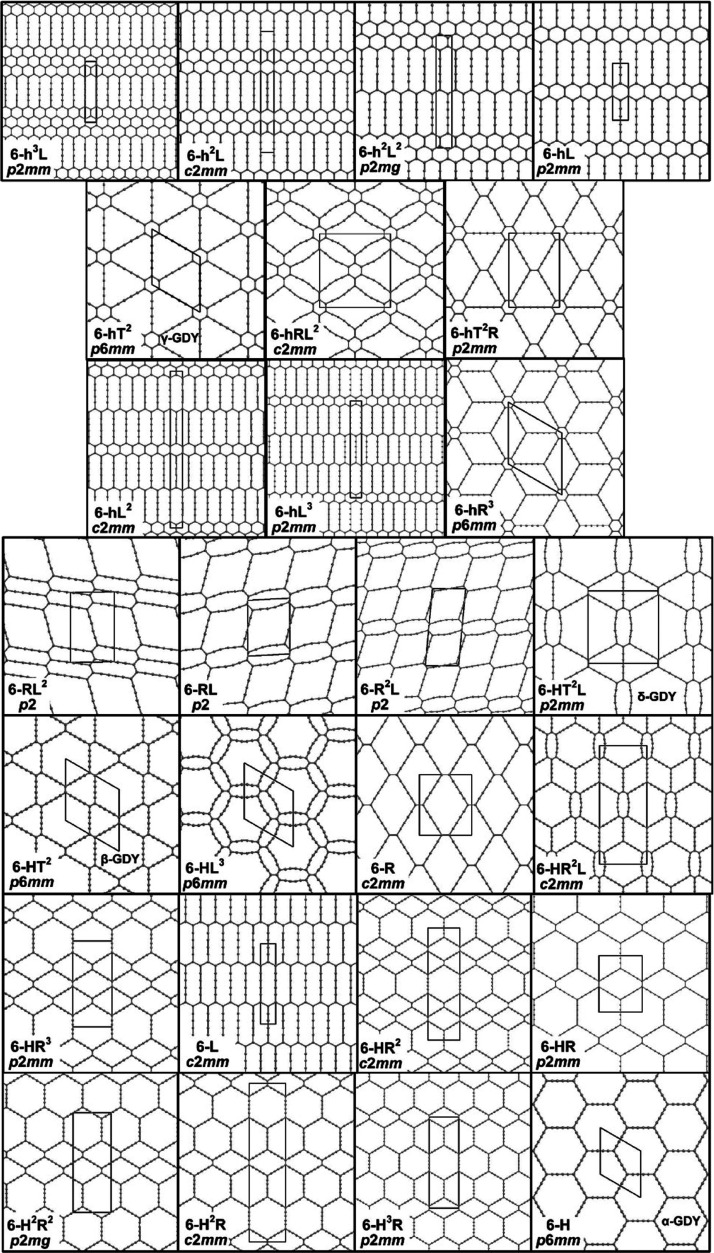
Representation of the 26 2D structures identified and investigated in this work. The unit cells and the plane groups are indicated. α-, β-, γ-, and δ-GDY are also labeled.

We developed an approach to generate and classify all possible GDY 2D structures. A similar enumeration problem was faced in the generation of graphane isomers; see ref (55). We considered stable graphene derivatives by inserting linear diacetylene (C_4_) groups for a given number of 3-coordinated carbon atoms (sp^2^ like) per primitive cell. We limited the analysis to a maximum of eight sp^2^ carbon atoms per primitive cell to avoid too large cells and to focus on structures that are more likely to be experimentally synthesized. This limit is enough to find all previously reported and many other GDY-like structures. Our approach is based on removing edges from the graphene structure and substituting them with linear diacetylenic units. The starting set of honeycomb layers with deleted edges was made of about 40,000 structures with a maximum of eight carbon atoms per primitive cell (four times that of the original cell). With the help of the topological classification tools in ToposPro, we extracted 332 topologically distinct patterns containing 6-membered rings with one or more missing edges. Geometrical considerations led to the possibly derived hexagons with deleted edges (from one to six), as shown in Figure 1. For each configuration, there is a “dual” one with reverse deleted edges, leaving 12 possible distinct patterns. In Figure 1 on the left, we show the eight patterns that after the introduction of C_4_ edges will not allow the closure of the 6-membered ring without a large angular distortion (see the detail for one case). Only the four remaining configurations shown in Figure 1 on the right allow expansion by the insertion of C_4_ linear structures without substantial distortion, that is, keeping all the angles around 120°. These four configurations and the original unaltered aromatic ring constitute five building blocks, here called as h,H,R,T,L (small and large hexagon h and H, respectively, rhombus R, triangle T, and line L), as suggested by Park et al.^17^ From the 332 patterns, we extracted only those containing some of the five possible building blocks that allow to tile the plane without a large distortion, obtaining 26 structures that are GDY-like (of which 17 are new), as described in Figure 2 and Table 1. Due to the “dual” properties of L/R and h/H, the 26 structures can be grouped in 12 couples plus a self-dual layer. Based on this classification, structures are called as 6-h^*n*^L^*m*^T^*o*^R^*p*^H^*q*^, where 6 represents the number of carbon atoms along the longest edge and the superscript on the building block symbols represents the number of each block appearing in a primitive unit cell (e.g., the primitive cell of *c*2*mm* 6-h^2^L contains two small C_6_ h-rings and one C_14_ L-ring, and all the edges are either between two or six carbon atoms).

**Table 1:** Summary of the 26 Structures Investigated Here: Reporting Name, Relative Energy with Respect to Graphene, Plane Group, Pearson Symbol, sp/sp^2^ Ratio, and Electronic Characters (Band Gap Reported for Semiconductors)^a^

name	rel. energy kcal/mol	plane group	Pearson symbol	sp/sp^2^ ratio	electronic character (PBE0)	refs
6-h^3^L	14.15	*p*2*mm*	oP12	0.50	metal	this work
6-h^2^L	16.81	*c*2*mm*	oS20	0.66	metal	this work
6-h^2^L^2^	20.55	*p*2*mg*	oP16	1.00	metal	this work
6-hL	20.59	*p*2*mm*	oP8	1.00	metal	this work
6-hT^2^ γ-GDY	21.13	*p*6*mm*	hP18	2.00	B.G. = 1.63 eV	(16−18)
6-hRL^2^	22.65	*c*2*mm*	oS48	2.00	0 B.G.	this work
6-hT^2^R	22.69	*p*2*mm*	oP28	2.50	B.G. = 0.83 eV	(17,18)
6-hL^2^	23.11	*c*2*mm*	oS28	1.33	metal	this work
6-hL^3^	24.06	*p*2*mm*	oP20	1.50	metal	this work
6-hR^3^	24.07	*p*6*mm*	hP32	3.00	0 B.G.	(17,18)
6-RL^2^	24.73	*p*2	mP22	2.66	0 B.G.	this work
6-RL	24.73	*p*2	mP16	3.00	0 B.G.	this work
6-R^2^L	25.03	*p*2	mP26	3.33	0 B.G.	this work
6-HT^2^L δ-GDY	25.07	*p*2*mm*	oP36	3.50	B.G. = 0.18 eV	this work
6-HT^2^ β-GDY	25.40	*p*6*mm*	hP30	4.00	B.G. = 1.14 eV	(16−18)
6-HL^3^	25.41	*p*6*mm*	hP32	3.00	0 B.G.	(18)
6-R	25.48	*c*2*mm*	oS20	4.00	0 B.G.	(16−18)
6-HR^2^L	25.85	*c*2*mm*	oS80	4.00	0 B.G.	this work
6-HR^3^	26.11	*p*2*mm*	oP44	4.50	0 B.G.	this work
6-L	26.18	*c*2*mm*	oS12	2.00	metal	(16,18)
6-HR^2^	26.30	*c*2*mm*	oS68	4.66	0 B.G.	this work
6-HR	26.63	*p*2*mm*	oP24	5.00	0 B.G.	(17,18)
6-H^2^R^2^	26.63	*p*2*mg*	oP48	5.00	0 B.G.	this work
6-H^2^R	26.92	*c*2*mm*	oS76	5.33	0 B.G.	this work
6-H^3^R	27.05	*p*2*mm*	oP52	5.50	0 B.G.	this work
6-H α-GDY	27.39	*p*6*mm*	hP14	6.00	0 B.G.	(16−18)

a In the last column, the reference number of previous papers investigating the same structure is reported.^16−18^

### 3.2 Relative Energies of 2D Crystals

**Figure 3:**
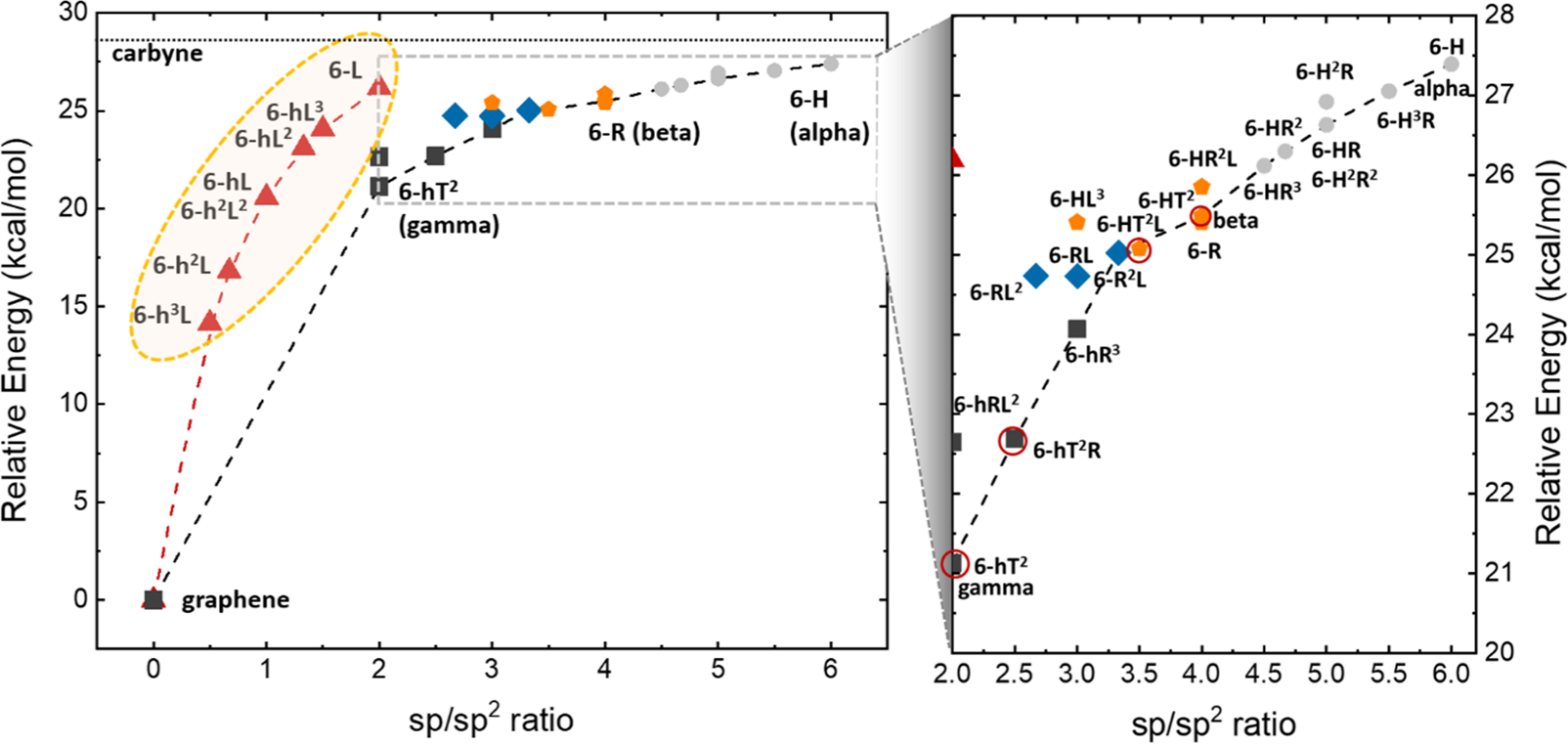
Plots of the DFT-computed relative energies of the 26 structures investigated here with respect to graphene as a function of sp–sp^2^ ratios. In the first panel, the two distinct trends are analyzed, while in the second panel, the members of the second trends are collected in different classes, depending on the peculiar topology identified. The seven metallic systems are all in the left panel and grouped in the elongated ellipsoid, while semiconductors with finite band gaps are circled in red. As a term of comparison, the computed energy of the 1D infinite carbyne is reported as an asymptotic limit of the curve (28.41 kcal/mol).

After the full geometry optimization of the 26 structures and of the reference 2D graphene structure, we investigated their stability by calculating the relative energy per carbon atom with respect to graphene
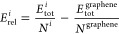
where *E*_tot_ is the DFT-computed total energy and *N* is the number of atoms in the unit cell for the *i*th structure and graphene (*N*^graphene^ = 2). This value of *R*_rel_ gives the relative cohesive energy per carbon atom and allows us to identify the most stable structures. Relative energy values are plotted as a function of the sp/sp^2^ ratio, calculated as the ratio of sp and sp^2^ carbon atom numbers in the unit cell (see Figure 3). This ratio ranges from 0 in the case of graphene up to 6 in the case of α-GDY, the largest values possible for the periodic 2D hybrid sp/sp^2^ carbon nanostructures investigated here where the sp domains are formed by diacetylenic units. The numerical values of the total and relative energies, sp/sp^2^ ratios, and layer densities are reported for all the structures in the Supporting Information (Table S2).

The increasing amount of sp carbon atoms with respect to graphene (i.e., a larger sp/sp^2^ ratio) increases the energy of the whole system, consistently with chemical intuition. However, in Figure 3, two different trends can be identified. The first trend (red line) is continuous and smooth and contains all the structures formed by different combinations of 6-atom hexagons (h) and line polygons (L) and, namely, 6-h^3^L, 6-h^2^L, 6-hL, 6-h^2^L^2^, 6-hL^2^, 6-hL^3^, and 6-L and graphene (6-h). The lowest-energy structure is 6-h^3^L, formed by graphene ribbons having a width of three aromatic units and interconnected by diacetylenic bridges. 6-h^2^L and 6-hL formed by graphene ribbons having a width of two and one aromatic units, respectively, and interconnected by diacetylenic bridges show an increase in relative energy. Hence, by reducing the width of graphene ribbons (i.e., as far as we progressively move away from the graphene limit), an increase in the relative energy is observed. Similarly, a further increase in energy is found in 6-hL^2^ and 6-hL^3^ which have, as in 6-hL, graphene nanoribbons of width 1 but which are interconnected by two and three L polygons (bridges of two and three diacetylenic units), respectively. Therefore, both the number of condensed aromatic hexagons (width of graphene ribbons) and the spacing between the graphene ribbons (h polygons) set by the number of diacetylenic domains (L polygons) modulate the relative energy of these systems with respect to graphene. The maximum energy is reached in 6-L where no h units are present at all. Considering, therefore, the general trend given by structures 6h^*n*^L^*m*^ in which graphene nanoribbons of width “*n*” are connected by “*m*” diacetylenic units, the lower energy is found by increasing *n* and decreasing *m*, clearly tending closer and closer to the graphene case. Interestingly, these two parameters seem to have similar weight; in fact, the same energy is obtained in 6-hL with the smallest graphene ribbon and single diacetylenic unit and in 6-h^2^L^2^ in which the increase in energy given by doubling the diacetylenic units is counterbalanced by doubling the graphene ribbon width. The structures of these groups have been investigated in a recent work with the name “grazynes”.^56^ Our analysis underlines a clear trend in the energy that can be easily generalized for similar structures for predictive purposes.

A different trend in Figure 3 is indicated by the green curve. In this group, we find the widely studied polymorphs of GDY, usually labeled as α-GDY (structure 6-H), β-GDY (structure 6-HT^2^), and γ-GDY (structure 6-hT^2^). γ-GDY has gathered more attention in the recent years, also from the experimental point of view. Among all the structures belonging to this trend, it has the lowest energy with respect to graphene, consistently with its low sp/sp^2^ ratio (sp/sp^2^ = 2). On the other hand, α-GDY is the highest-energy structure among all of those which are investigated here, again consistently with the highest sp/sp^2^ ratio (sp/sp^2^ = 6). β-GDY with sp/sp^2^ = 4 is in between these two limiting cases.

Apart from these widely studied polymorphs, the structures belonging to this second group can be further classified based on their topology and structure. The description of their geometry is based on their building units, similarly to the method proposed by Park et al.^17^ As shown in Figure 3, the four lowest-energy structures 6-hT^2^ (γ-GDY), 6-hRL^2^, 6-hT^2^R, and 6-hR^3^ form one subgroup themselves, since they all share the presence of h polygons in their geometry, that is, a last reminiscence of the graphene structure. On the other hand, the highest-energy structures 6-HR^3^, 6-HR^2^, 6-HR, 6-H^2^R^2^, 6-H^2^R, 6-H^3^, and 6-H(α-GDY) form another subgroup, and they can be all described as α-GDY ribbons connected with different widths and interconnections, tending indeed toward the upper limit of α-GDY.

In the intermediate case, two clusters of structures can be identified: one collects structures 6-HL^3^, 6-HT^2^L, 6-R, 6-HR^2^L, and 6-HT^2^ (β-GDY) and the other one collects the structures 6-RL, 6-RL^2^, and 6-R^2^L. The members in the first group are all characterized by the presence of isolated α-GDY units (i.e., large 30-atom hexagons H with all sizes characterized by diacetylenic units), while in the second group, the systems are structurally peculiar, since they are formed only by units which can be described as rhombus R and lines L according to the definition in Figure 1. It should be noticed that in many of the structures containing L units, the diacetylenic bridges are usually bent and not linear, as also found in the calculations by Belenkov et al.^16^ Moreover, the energy values of 6-HR/6-H^2^R^2^ and 6-hL/6-h^2^L^2^ seem to overlap between each other, even if they are not exactly the same values. Such close values could be due to the fact that they contain the same structural units (H and R for the former two and h and L for the latter) in the same ratio, suggesting the role of topology in determining the energetic and electronic behavior of these systems.

The energy trend can be summarized as follows: (1) As expected, the relative energy with respect to graphene increases for an increasing sp/sp^2^ ratio. (2) The closer the structural resemblance to graphene, the lower the relative energy of the 2D structures. This is clearly shown in crystals made of graphene ribbons having diacetylenic-connected bridges: the more we approach the graphene structures (the lower the sp/sp^2^ ratio), the lower the relative energy. (3) Considering the other trends, the lowest-energy structures are those where the graphene h unit (hexagon of sp^2^ carbon) is still present in the structure. (4) The energy increases when the number of α-GDY units (H hexagon) increases up to the limiting case of 2D α-GDY.

These trends in relative energy are consistent with the cohesive energies reported by Park et al.^17^ and with the sublimation energy given by Belenkov et al.^16^ by considering that a larger relative energy corresponds to a lower cohesive and sublimation energy. Our work consistently with Park et al.^17^ shows that α-GDY has the lowest stability, while γ-GDY is the most stable. On the other hand, the semiempirical calculations by Belenkov et al.^16^ predict the largest sublimation energy (i.e., lower relative energy) for the system called γ2, which corresponds to 6-L in our work. For 6-L, we find a larger relative energy than γ-GDY even if they share the same sp/sp^2^ ratio value. Our results show a general qualitative agreement with ref (18). Relative energy (here with respect to graphene and in ref (18) with respect to γ-graphyne) increases with decreasing carbon densities (i.e., increasing sp/sp^2^ ratio). Our work includes some structures already investigated in the literature and several novel ones, thus revealing the wide range of possibilities available in the design of 2D sp–sp^2^ carbon systems.

As a term of comparison, we carried out a simulation, adopting the same level of theory, of the 1D carbyne chain, the limiting case in which the sp/sp^2^ tends to infinity, and we found a relative energy of 28.41 kcal/mol, which is, as expected, the largest energy value reported in this paper and should be intended as an asymptotic limit when increasing the sp/sp^2^, as can be seen in Figure 3. Indeed, it can be considered as the limiting case for all the possibly generated 2D hybrid sp/sp^2^ carbon structures.

In Figure 3, seven structures that show a metallic behavior are grouped with an elongated ellipsoid, while the finite band gap semiconducting crystals are circled in red. All the other 2D carbon systems are zero-band gap semiconductors, as discussed in the following chapter.

### 3.3 Electronic Properties: Band Structure and DOS

**Figure 4:**
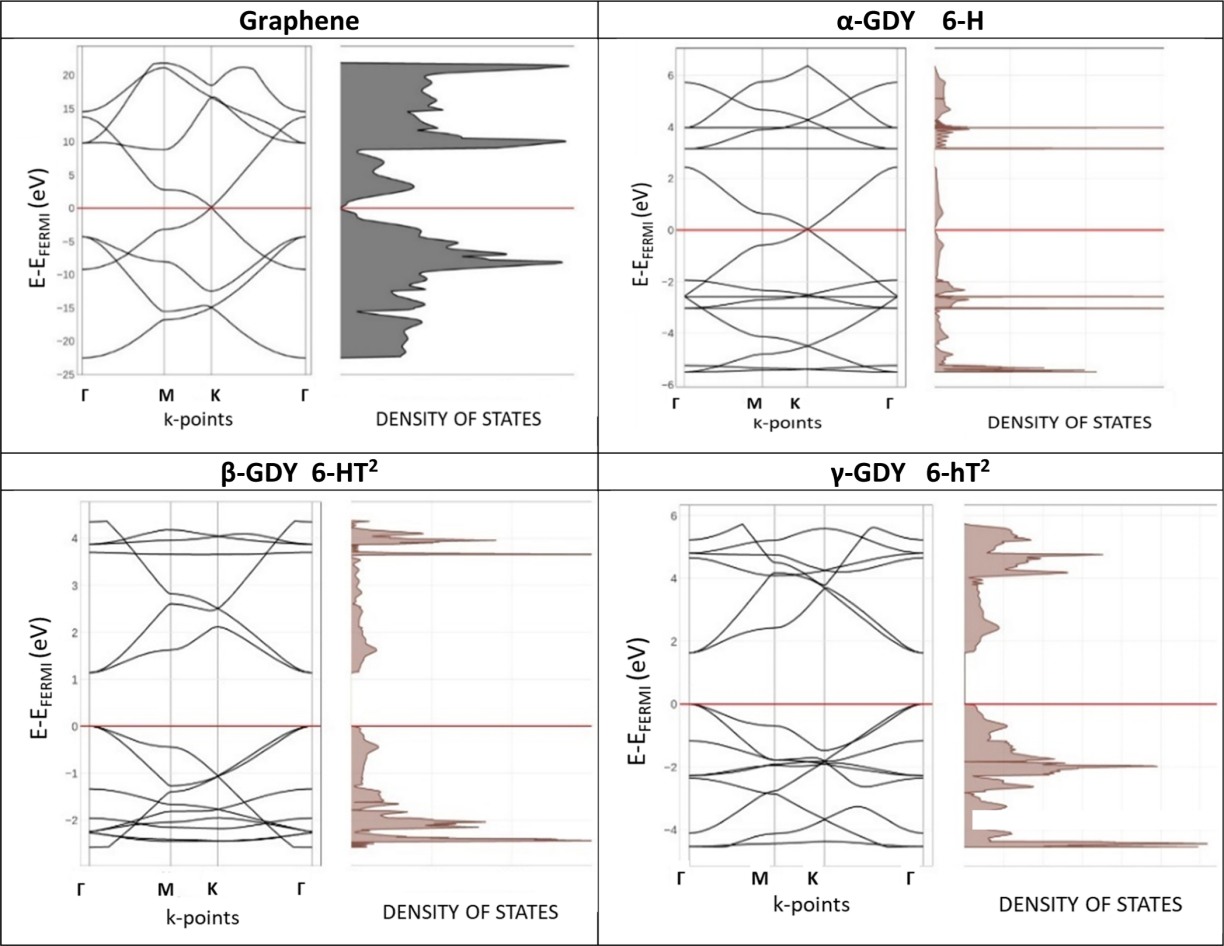
Comparison of DFT-computed band structures and DOS of graphene and α-, β-, and γ-GDY polymorphs.

We discuss here how topological elements affect the band structure and the electronic properties in GDY crystals, on the basis of their peculiar crystal structure. In Figure 4, we compare the band structure of graphene with that of the three widely investigated α-, β-, and γ-polymorphs of GDY. α-GDY shows a very similar band structure to that of graphene, with the occurrence of Dirac cones at the K-point, which makes both of them zero-gap semiconductors or semimetals, in agreement with previous studies.^21,22^ Graphene and α-GDY have the same crystal structure and layer group (*p*6*mm*), differing only in the number of carbon atoms (2 and 14 in graphene and α-GDY, respectively), highlighting the topology dependence of the band structure. In α-GDY, we also observed the appearance of both occupied and empty flat bands, another typical topology-dependent feature found for 2D COFs.^23−26^

On the other hand, β-GDY and γ-GDY are finite-gap semiconductors, showing a band gap of 1.14 and 1.63 eV, respectively. Their structures are similar: based on the algorithm we adopted to build the structures, β-GDY and γ-GDY are indeed dual ones to the other (see also Figures 1 and 2), respectively, 6-HT^2^ and 6hT^2^, demonstrating the topology dependence of the band structure.

**Figure 5:**
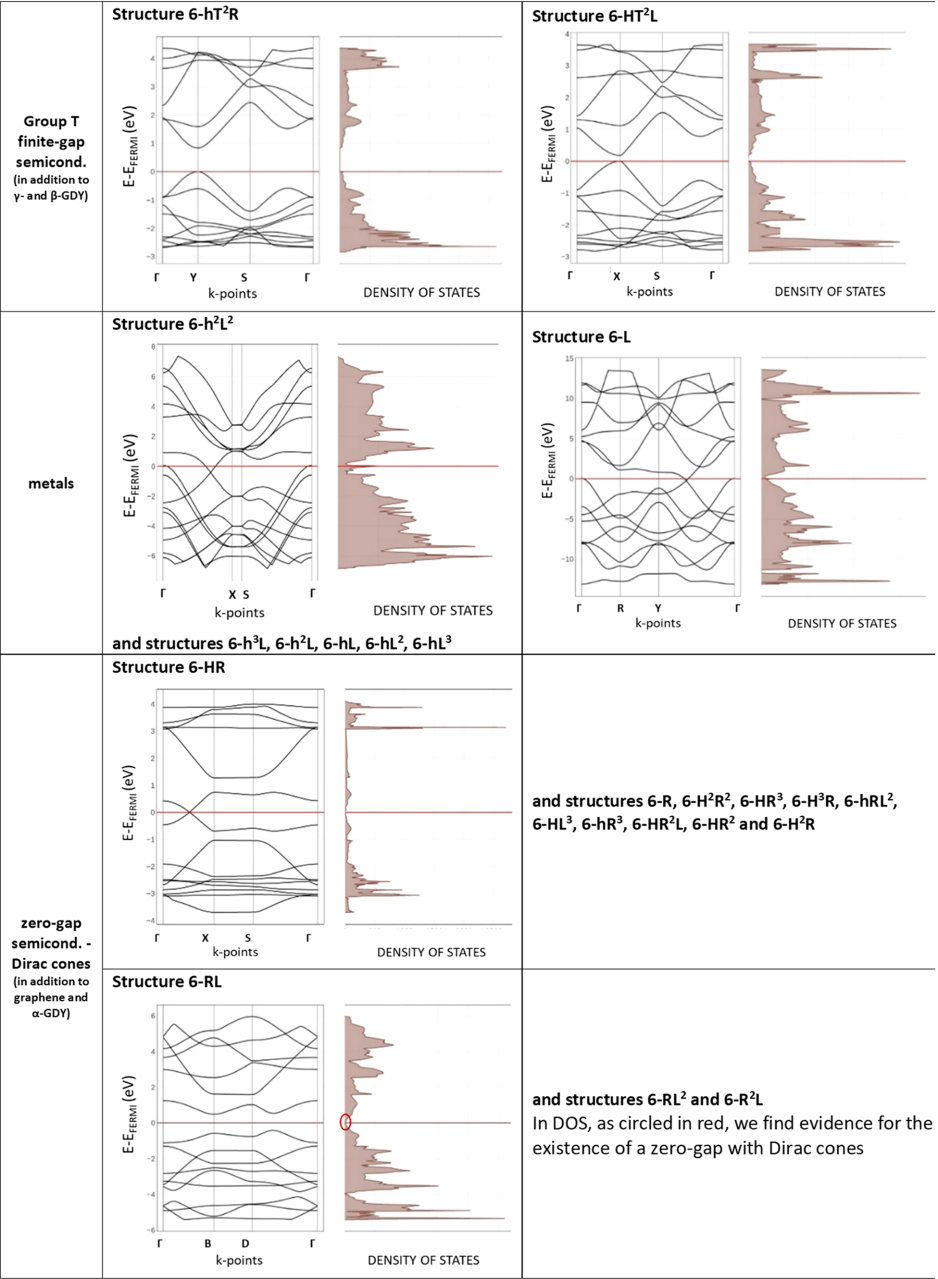
Comparison of DFT-computed band structures and DOS of all the other structures investigated here.

Further results can be obtained by extending the analysis to all the systems investigated here. The band structure and DOS of all these structures can be classified into three classes: finite-gap semiconductors (such as β- and γ-GDY), metals, and zero-gap semiconductors (see Figure 5). For each class, the band structure and DOS of only one representative structure are reported, while a complete table for all the geometries investigated is reported in the Supporting Information (Table S1).

Finite-gap semiconductors include β- and γ-GDY and only two other structures, 6-hT^2^R and 6-HT^2^L, presenting a finite gap of 0.8327 and 0.1789 eV, respectively. These geometries are dual ones with respect to the other, as for β- and γ-GDY (6-HT^2^ and 6-hT^2^, respectively; see Figure 1). Among the 26 structures investigated here, these are the only ones containing a T-shaped unit (see Figure 1), suggesting that T units would induce electronic effects, leading to the occurrence of a band gap.

The second class is formed by metallic systems and include structures made of graphene ribbons interconnected by diacetylenic groups (here called as hL), also described as grazynes.^56^ All these crystals (i.e., 6-h^3^L, 6-h^2^L, 6-hL, 6-h^2^L^2^, 6-hL^2^, 6-hL^3^, and 6-L) share the same trend in the band structure. They present a half-filled band, in a structure characterized by a Dirac cone immediately below the Fermi energy. In this class, we find also structure 6-L, the limiting case of a 2D crystal formed by line units only.

The third class, collecting the largest number of different geometries, is characterized by zero-gap semiconductors presenting Dirac cones at the Fermi energy. In this class, two subclasses with a slightly different behavior are presented: the first one, including structures 6-R, 6-HR, 6-H^2^R^2^, 6-HR^3^, 6-H^3^R, 6-hRL^2^, 6-HL^3^, 6-hR^3^, 6-HR^2^L, 6-HR^2^, and 6-H^2^R, collects 2D crystals that, from the point of view of the band structure, present a pattern similar to that of α-GDY, with a Dirac cone along one of the special directions in the BZ. The second subclass (RL) collects structures 6-RL, 6-RL^2^, and 6-R^2^L, which already formed a cluster in relative energy: for these three crystals, Dirac cones cannot be identified along the three main paths in the BZ but are located elsewhere in the Brillouin zone. The presence of Dirac cones is outlined by the linear behavior of the DOS close to the Fermi energy. Even for these structures sharing a peculiar topology (they are crystals formed only by rhombus R and line L units), 6-RL^2^ and 6-R^2^L are dual ones with respect to the other, and 6-RL is self-dual.

To support the significance of these results, for some structures, geometry optimization and band structure/DOS calculations have been repeated using the HSE06 functional together with the same 6-31G(d) basis set, in order to check the effect related to the functional choice. As also demonstrated in our previous work on y-GDY (6-hT^2^), this functional can indeed give a more accurate quantitative evaluation of the band gap, in agreement with benchmark GW-computed values, even if the qualitative trends are the same obtained with PBE0.^31^ The comparison between the band structure/DOS computed with these two functionals is reported in the Supporting Information (Table S1). As expected, HSE06 band gap values are lower: 1.11 versus 1.63 eV for 6-hT^2^ (y-GDY), 0.34 versus 0.83 eV for 6-hT^2^R, and 0.68 versus 1.14 eV for 6-HT^2^ (β-GDY); however, they still confirm that these structures are finite-band gap semiconductor. A discrepancy is found, however, for the 6-HT^2^L structure, showing a very small (0.18 eV) but finite band gap with PBE0, while it is predicted as a zero-band gap semiconductor with HSE06. This result shows that in the presence of very small band gaps, the choice of the theoretical method could play an important role.

These trends reveal the topology-related shape of the band structure and the metallic, finite- or zero-gap semiconductor behavior.

An inspection of the geometry of these structures allows further insights into how the building units (Figure 1) determine the behavior of the 2D carbon materials. A finite band gap can be the consequence of the presence of T units, while hexagonal units (h and H) are present in zero-band gap semiconductors with Dirac cones, as evidenced by the limiting case of graphene (h units only) and α-GDY (H units only). R units (see the case of 6-R) are similar to h and H, promoting the occurrence of zero-gap semiconducting behavior with Dirac cones, while L units, as in the case of 6-L (only L building blocks), occur in structures showing a metallic band structure. We can conclude that whenever h, H, or R units are present, there would be a tendency toward zero-gap semiconductors showing Dirac cones, and when L units are present, there would be a tendency toward metallic structures, while, on the other hand, T units are related to a gap opening between valence and conduction bands.

All metallic structures are formed by systems where only h and L units are present, and the effect of L seems to dominate that of h in affecting the band structure. Zero-gap structures all contain H and/or R, and for some of them, also, h and L units can be present: in any case, H/R units seem to dominate the behavior of the band structure. Finally, T units seem to dominate over all the other units in affecting the band structure, promoting a band gap. Interestingly, 6-HT^2^L represents a limiting case, showing a balance between the opposite effects of T and L units. The dominating effect seems to be related to the functional choice, since we find 0.18 eV for PBE0 and zero gap for HSE06.

For the systems already reported in the literature, our results are consistent and describe the same behavior.^17^ However, the band gap values are lower than the ones reported here for 6-HT^2^ and 6-hT^2^, probably due to the use of a pure GGA functional (PBE) with respect to the hybrid one used in our calculation. A discrepancy is found for the 6-hT^2^R structure, for which we predict a gap of 0.83 eV with PBE0 (and 0.34 eV with HSE06), while a zero-gap semiconductor is predicted by Park et al.^17^ This points out again that for semiconductive systems having a small band gap (i.e., below 1 eV), the choice of the functional could be relevant to predict the behavior of the materials in terms of their electronic structure.

Based on the role of the different h, H, R, L, and T units and considering the results for 6-HT^2^L with PBE0 and HSE06, the choice of the functional is relevant in predicting the smaller or larger dominating effect of the different units in affecting the band structure behavior. This is not a primary effect when large band gaps are present, but some peculiar cases could require more attention to the theoretical method.

## 4 Conclusions

The possibility to develop sp–sp^2^ carbon 2D materials by playing on the topology and connectivity of sp and sp^2^ domains or on their relative ratio is clearly appealing for both fundamental and applied research with possible outcomes in technology. Up to now, most of the experimental research on GDY systems focused on the development of proper synthesis techniques, while a huge amount of theoretical investigation has been focused on the properties of these materials. However, most of the literature focused on the γ polymorph of GDY and related systems (nanoribbons, molecular fragments, ...) or in some cases on the α- and β-GDY. Only a few other possible structures have been considered so far. There is ideally a wide range of possible sp–sp^2^ carbon materials, in the form of 2D crystals, that could be possible alternatives to γ-GDY.

We proposed a computational investigation aimed at the molecular design of new sp–sp^2^ carbon 2D crystals, focusing in particular on the importance of the structure and topology in modulating the relative energies and the band structure with respect to graphene. Our approach is able to predict all the possible sp–sp^2^ crystals as graphene derivatives. By restricting our search to all the sp–sp^2^ carbon crystals with a maximum number of eight sp^2^ carbon atoms per unit cell, we generated 26 2D crystals. DFT simulations under periodic boundary conditions have been carried out, revealing some peculiar trends both in relative energy and electronic properties, which can be described in terms of general topological effects.

In all the cases, an increase in the sp–sp^2^ carbon ratio produced an increase in relative energies with respect to graphene, with two peculiar trends. A first one is constituted by graphene stripes interconnected by diacetylenic bridges (grazynes), which have been also predicted to have a metallic behavior. The second trend collects 2D crystals (including α-, β-, and γ-GDY), which can be described in terms of common geometrical units formed by the carbon atoms, including h and H hexagons, L lines, R rhombus, and T triangles. Describing the crystals in terms of these units allowed us to rationalize both relative energies and the band structure: the higher the similarity to graphene units (i.e., h), the lower the relative energies; on the other hand, H units increase the relative energies up to the limiting case of α-GDY and are a characteristic of zero-gap semiconductors with Dirac cones.

These different units can play a role in determining the electronic behavior of the material: triangular T units are indeed the structural/topological factor which promotes semiconductive materials with a finite band gap; on the other hand, L units would promote metallic structures, while h, H, and R units tend to induce zero-gap semiconducting behavior with Dirac cones. As a general rule, in structures formed by different units, L is found to have a larger effect than h, while H and R dominate over L, and finally, T seems to dominate all over the other units, even if these relative effects have been shown to have a non-negligible dependence on the DFT functional choice, in particular for some peculiar structures. Therefore, we demonstrated that the local topology is strongly responsible for the metallic/semiconductive behavior, while the formation of long conjugation pathways, along which a larger/lower conjugation can occur, seems to play only a minor role, in agreement with the behavior of γ-GDY fragments.^32^

These findings give a relevant indication to properly develop new semiconductive sp–sp^2^ carbon materials, since they are able to give general and simple topological rules to design new systems built as a proper combination of simple building blocks (h, H, R, L, and T units), where the electronic properties are properly tailored by precisely controlling their topology. This will offer many outcomes in view of applications and some insights into engineering new carbon nanostructured materials with tailored properties.

## Supporting Information Available

Crystallographic information (CIF)
Computed band structures, density of states (DOS), and crystallographic information for all the 26 2D GDY-based crystals investigated in the work (PDF)

## Supplementary Material

jp1c04238_si_001.cif
