# Supplementary material for: Designing All Graphdiyne Materials as Graphene Derivatives: Topologically Driven Modulation of Electronic Properties
Source: J Phys Chem C Nanomater Interfaces. 2021 Jul 15;125(33):18456–66. doi: 10.1021/acs.jpcc.1c04238 (PMC8404194; doi:10.1021/acs.jpcc.1c04238)
Supplement: Supplementary file 2 — jp1c04238_si_002.pdf [file jp1c04238_si_002.pdf]

Supporting Informations

Designing all graphdiyne materials as graphene derivatives: topologically driven modulation of electronic properties

Patrick Serafini[1], Alberto Milani[1*], Davide M. Proserpio[2,3], Carlo S. Casari[1*]

[1]Dipartimento di Energia, Politecnico di Milano, via Ponzio 34/3, Milano, Italy
[2]Dipartimento di Chimica, Università degli Studi di Milano, 20133 Milano, Italy
[3]Samara Center for Theoretical Materials Science (SCTMS), Samara State Technical University, Samara 443100, Russia

*Corresponding authors: alberto.milani@polimi.it; carlo.casari@polimi.it

Table S1: *Computed band structures and density of states (DOS), with a sketch of the structure for each of the analysed 2D crystal, are reported. For some specific cases, a comparison between band structures and DOS calculated with PBE0 and HSE06 exchange-correlation density functionals is reported.*

Names	Band Structure + DOS (PBE0)	Band Structure + DOS (HSE06)
**Graphene**	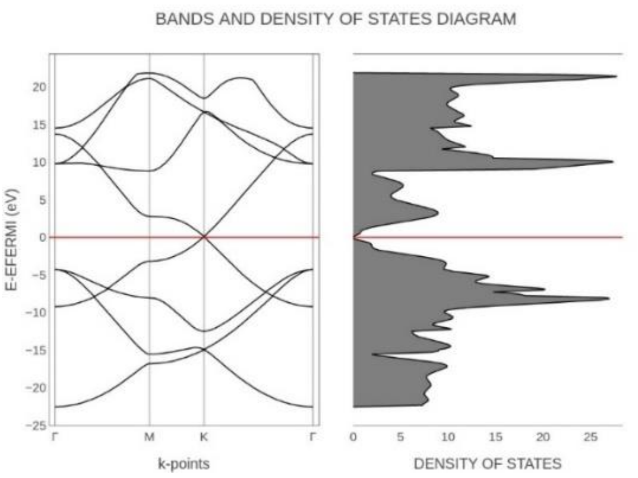	

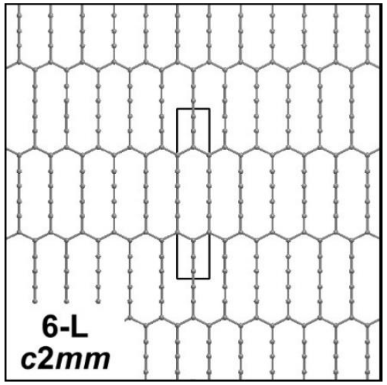
6-L
c2mm

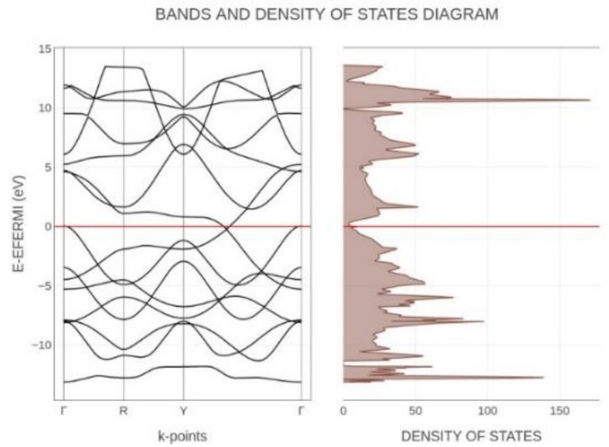
BANDS AND DENSITY OF STATES DIAGRAM
E-EFERMI (eV)
Γ R Y Γ
k-points
DENSITY OF STATES

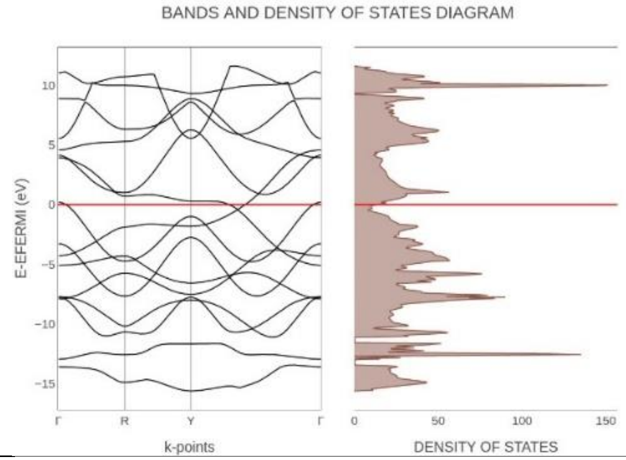
BANDS AND DENSITY OF STATES DIAGRAM
E-EFERMI (eV)
Γ R Y Γ
k-points
DENSITY OF STATES

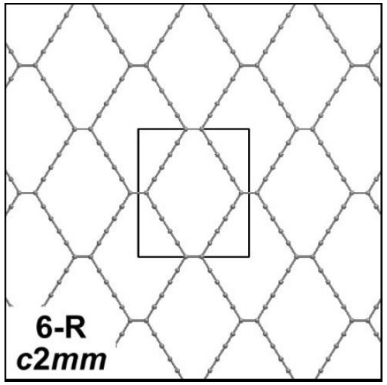
6-R
c2mm

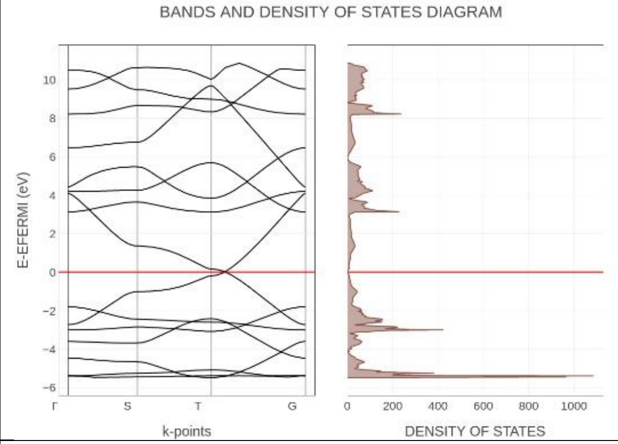
BANDS AND DENSITY OF STATES DIAGRAM
E-EFERMI (eV)
Γ S T G
k-points
DENSITY OF STATES

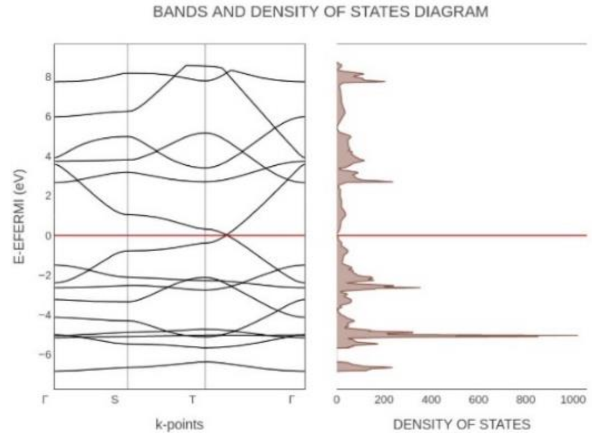
BANDS AND DENSITY OF STATES DIAGRAM
E-EFERMI (eV)
Γ S T Γ
k-points
DENSITY OF STATES

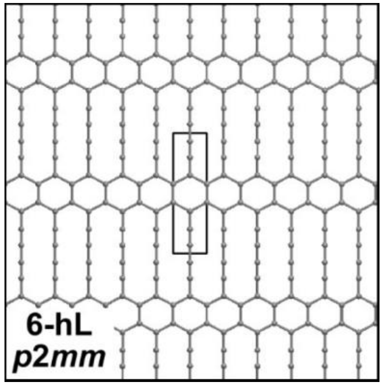
6-hL
p2mm

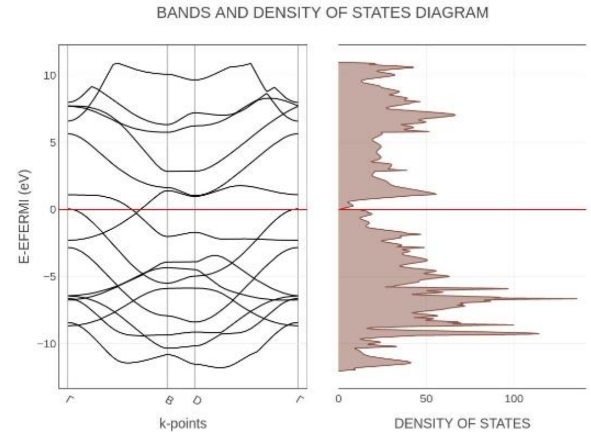
BANDS AND DENSITY OF STATES DIAGRAM
E-EFERMI (eV)
Γ B D Γ
k-points
DENSITY OF STATES

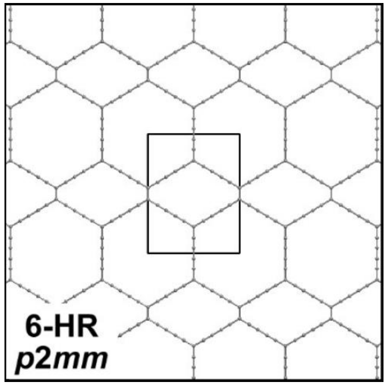
6-HR
p2mm

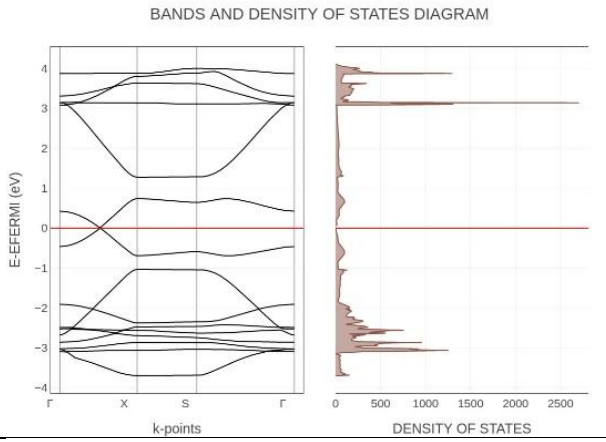
BANDS AND DENSITY OF STATES DIAGRAM
E-EFERMI (eV)
Γ X S Γ
k-points
0 500 1000 1500 2000 2500
DENSITY OF STATES

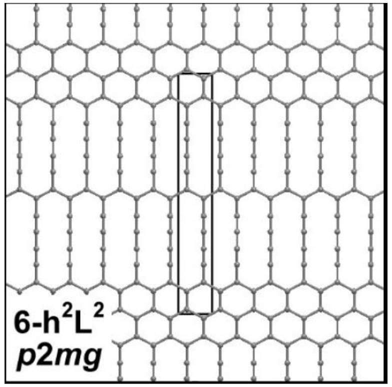
6-h^{2}L^2
p2mg

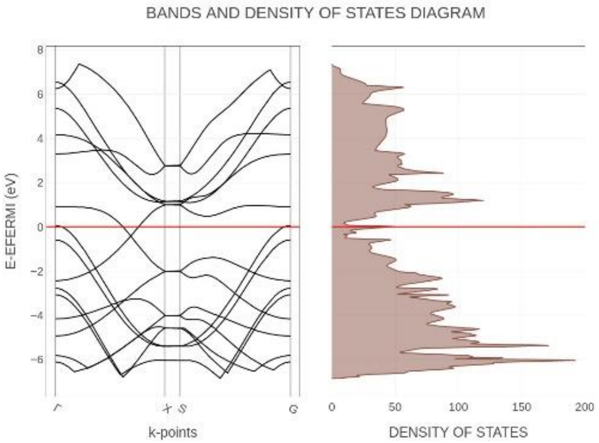
BANDS AND DENSITY OF STATES DIAGRAM
E-EFERMI (eV)
k-points
0 50 100 150 200
DENSITY OF STATES

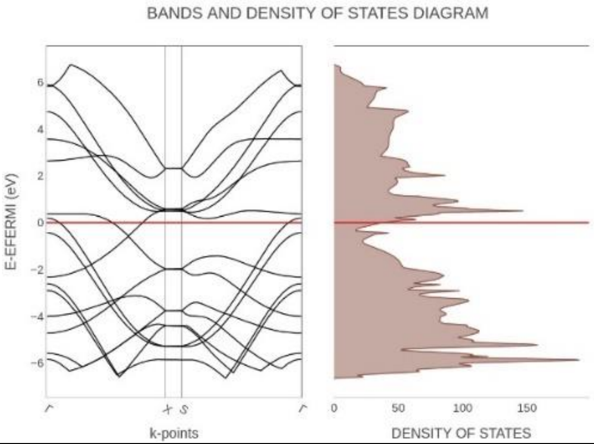
BANDS AND DENSITY OF STATES DIAGRAM
E-EFERMI (eV)
k-points
0 50 100 150
DENSITY OF STATES

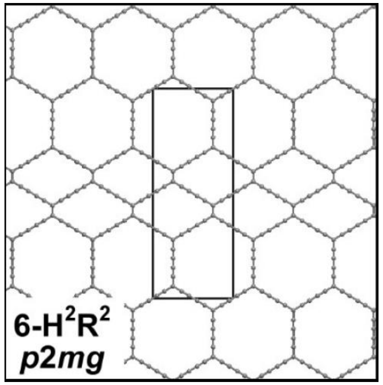
6-H^{2}R^2
p2mg

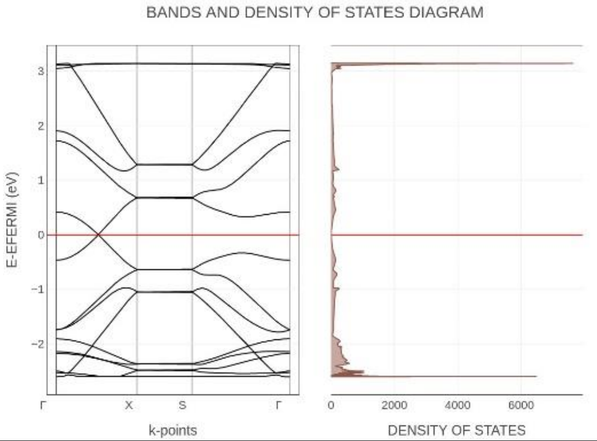
BANDS AND DENSITY OF STATES DIAGRAM
E-EFERMI (eV)
Γ X S Γ
k-points
0 2000 4000 6000
DENSITY OF STATES

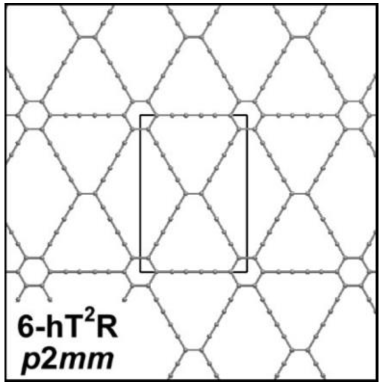
6-hT^{2}R
p2mm

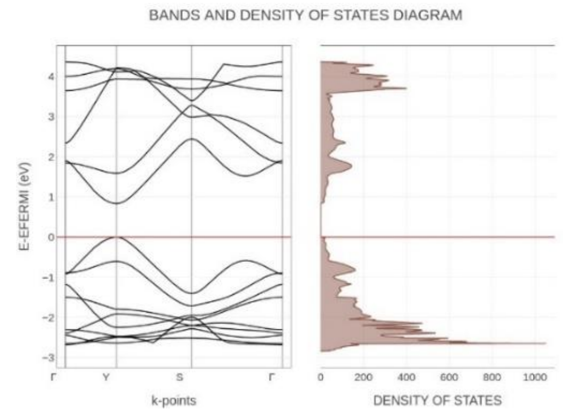
BANDS AND DENSITY OF STATES DIAGRAM
E-EFERMI (eV)
Γ Y S Γ
k-points
DENSITY OF STATES

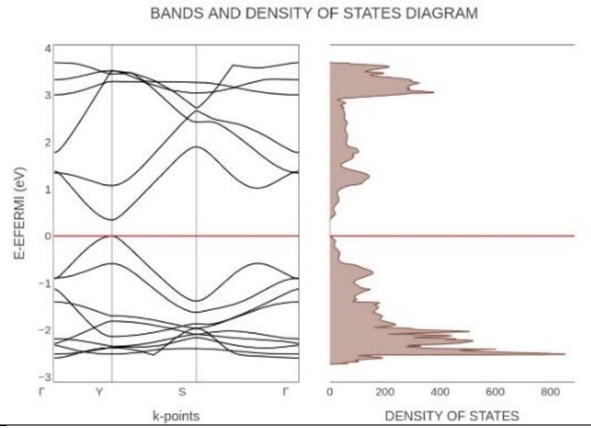
BANDS AND DENSITY OF STATES DIAGRAM
E-EFERMI (eV)
Γ Y S Γ
k-points
DENSITY OF STATES

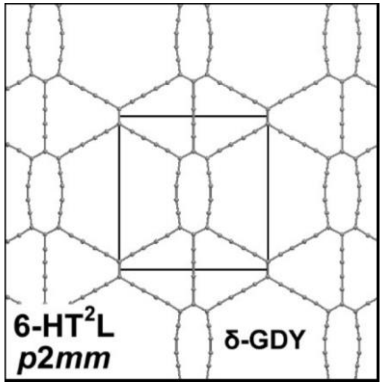
6-HT^{2}L
p2mm
δ-GDY

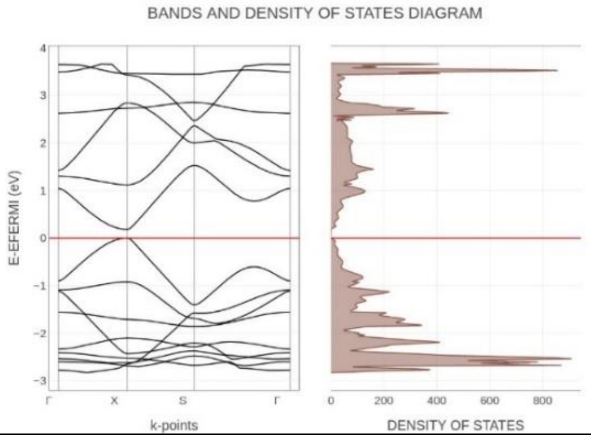
BANDS AND DENSITY OF STATES DIAGRAM
E-EFERMI (eV)
Γ X S Γ
k-points
DENSITY OF STATES

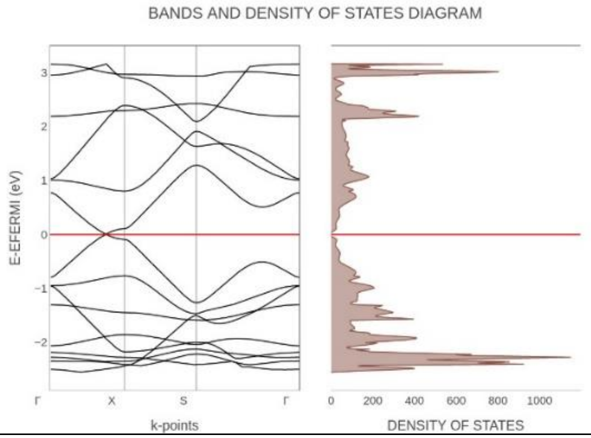
BANDS AND DENSITY OF STATES DIAGRAM
E-EFERMI (eV)
Γ X S Γ
k-points
DENSITY OF STATES

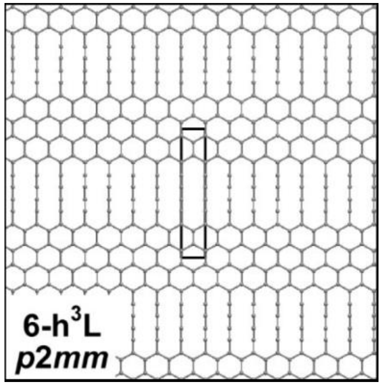
6-h^{3}L
p2mm

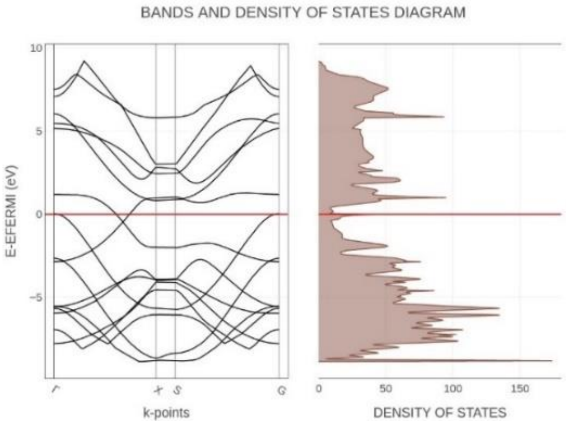
BANDS AND DENSITY OF STATES DIAGRAM
E-EFERMI (eV)
k-points
DENSITY OF STATES

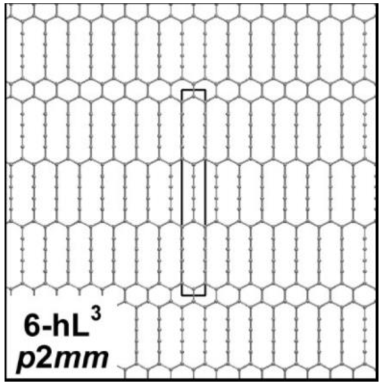6-hL^3<br>*p2mm*	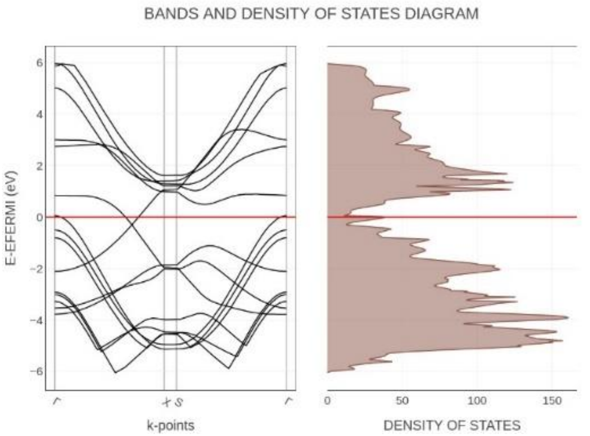BANDS AND DENSITY OF STATES DIAGRAM<br>E-EFERMI (eV)<br>k-points<br>DENSITY OF STATES	
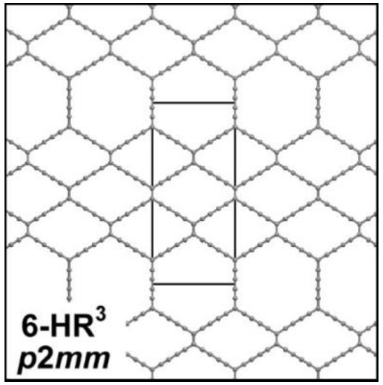6-HR^3<br>*p2mm*	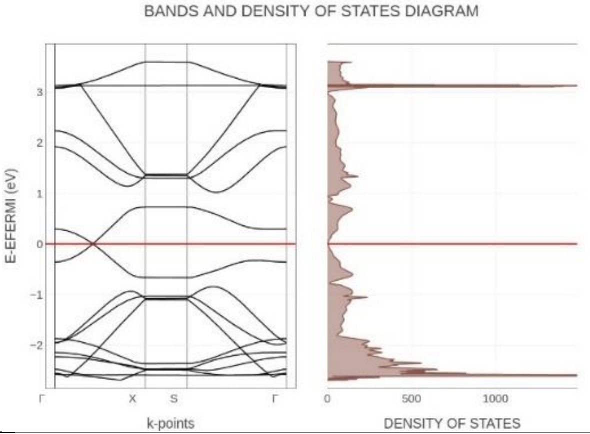BANDS AND DENSITY OF STATES DIAGRAM<br>E-EFERMI (eV)<br>k-points<br>DENSITY OF STATES	
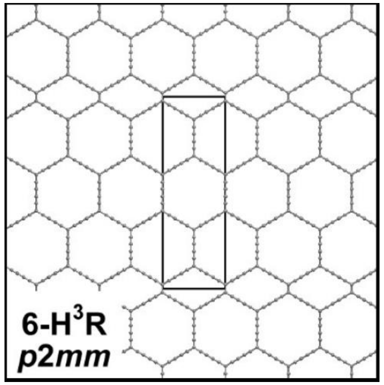6-H^{3}R<br>*p2mm*	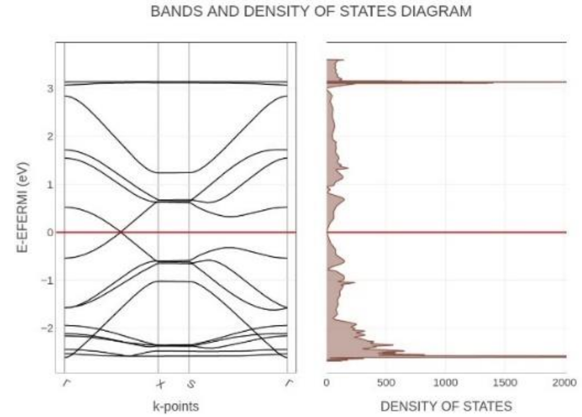BANDS AND DENSITY OF STATES DIAGRAM<br>E-EFERMI (eV)<br>k-points<br>DENSITY OF STATES	

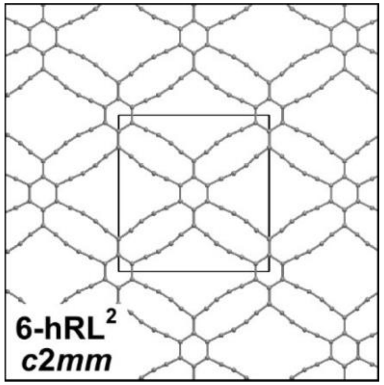6-hRL^2<br>*c2mm*	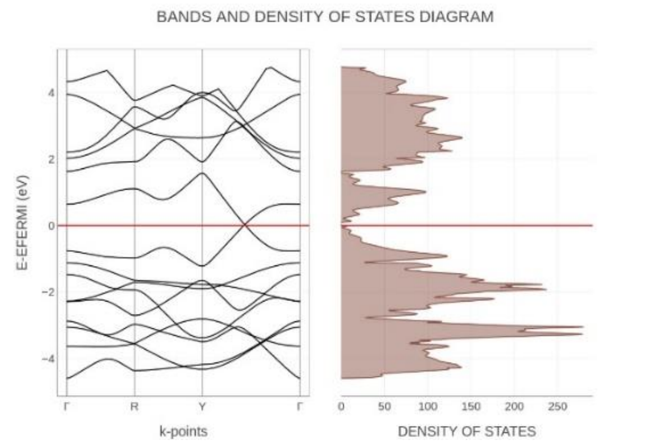BANDS AND DENSITY OF STATES DIAGRAM<br>E-EFERMI (eV)<br>Γ R Y Γ<br>k-points<br>DENSITY OF STATES	
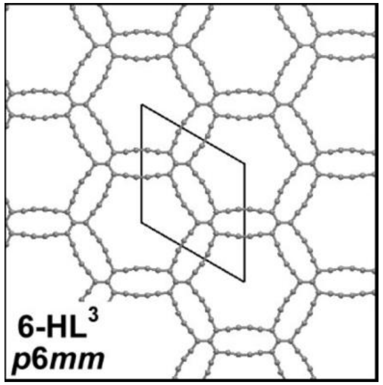6-HL^3<br>*p6mm*	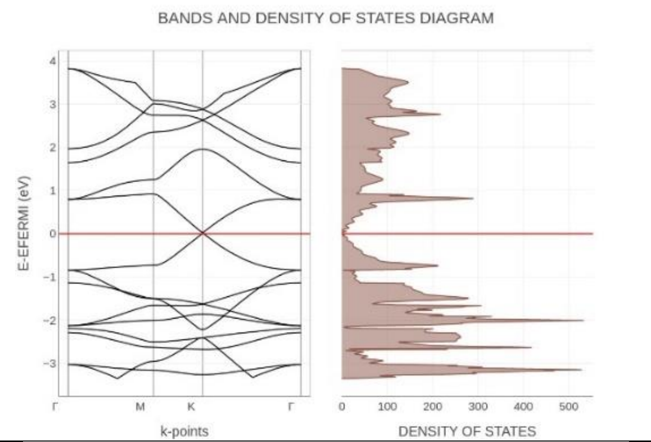BANDS AND DENSITY OF STATES DIAGRAM<br>E-EFERMI (eV)<br>Γ M K Γ<br>k-points<br>DENSITY OF STATES	
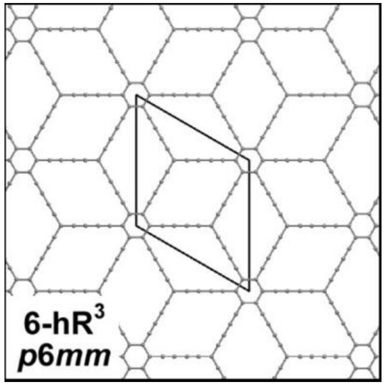6-hR^3<br>*p6mm*	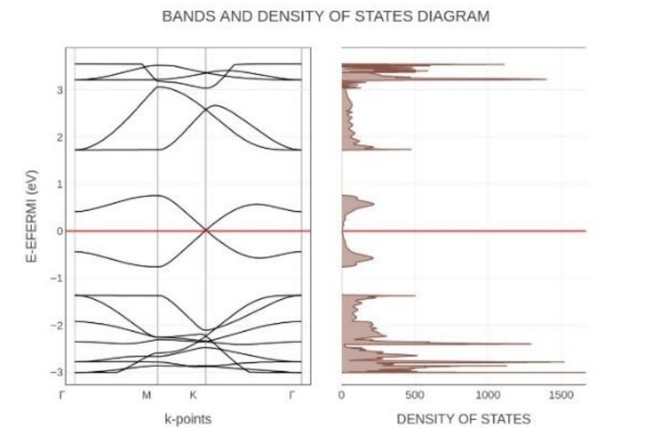BANDS AND DENSITY OF STATES DIAGRAM<br>E-EFERMI (eV)<br>Γ M K Γ<br>k-points<br>DENSITY OF STATES	

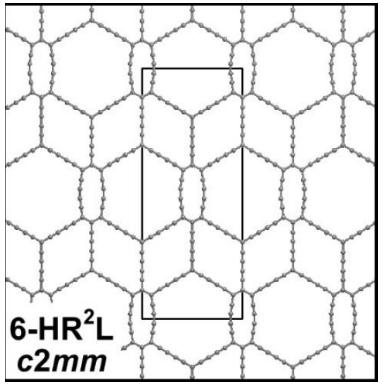6-HR²L c2mm	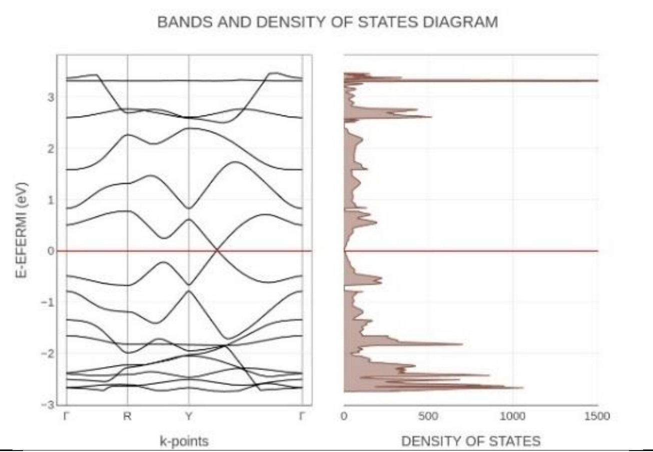BANDS AND DENSITY OF STATES DIAGRAM E-EFERMI (eV) k-points DENSITY OF STATES	
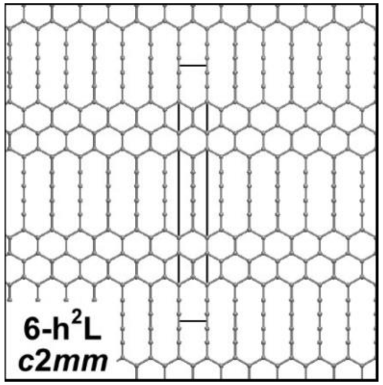6-h²L c2mm	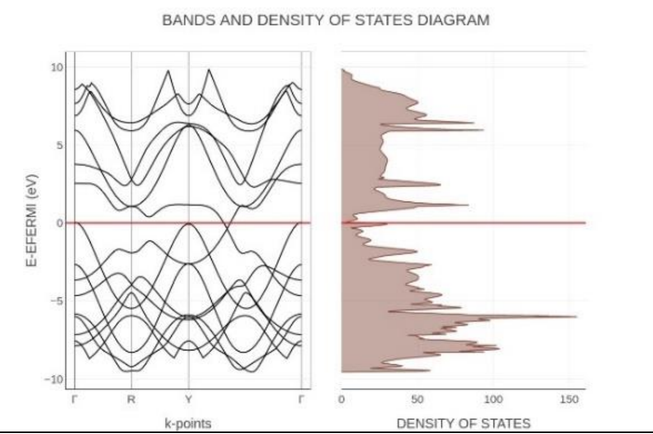BANDS AND DENSITY OF STATES DIAGRAM E-EFERMI (eV) k-points DENSITY OF STATES	
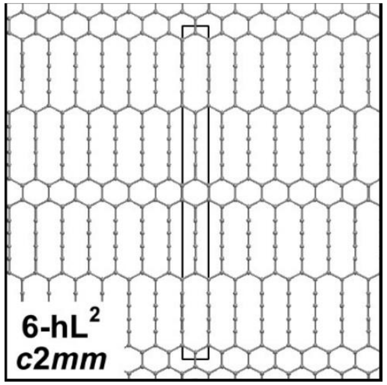6-hL² c2mm	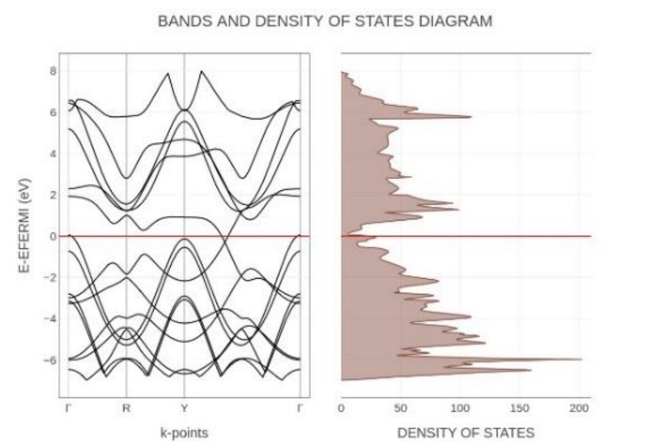BANDS AND DENSITY OF STATES DIAGRAM E-EFERMI (eV) k-points DENSITY OF STATES	

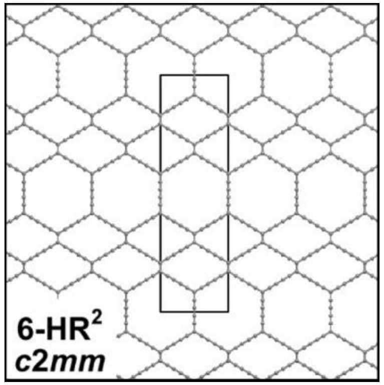
6-HR^2
c2mm

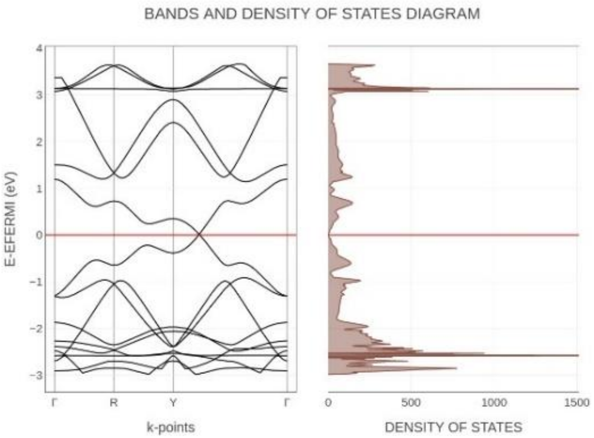
BANDS AND DENSITY OF STATES DIAGRAM
E-EFERMI (eV)
k-points
DENSITY OF STATES

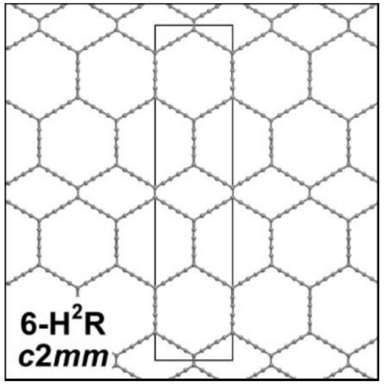
6-H^{2}R
c2mm

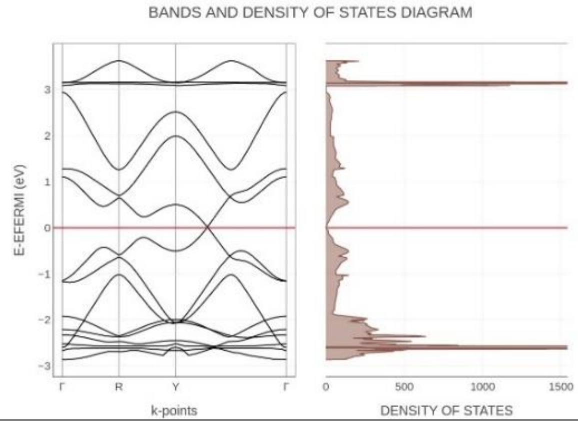
BANDS AND DENSITY OF STATES DIAGRAM
E-EFERMI (eV)
k-points
DENSITY OF STATES

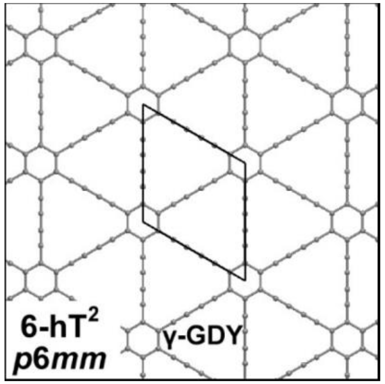
6-hT^2
p6mm
γ-GDY

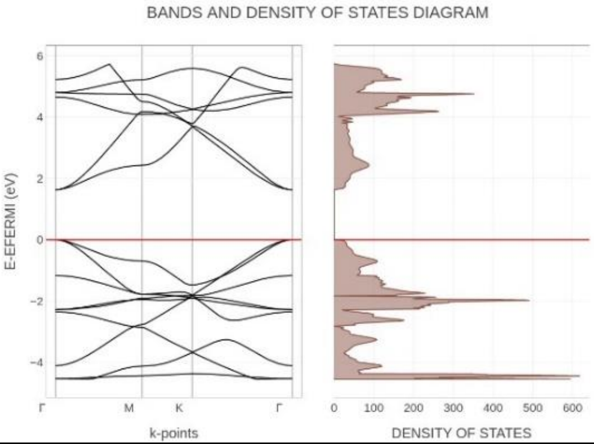
BANDS AND DENSITY OF STATES DIAGRAM
E-EFERMI (eV)
k-points
DENSITY OF STATES

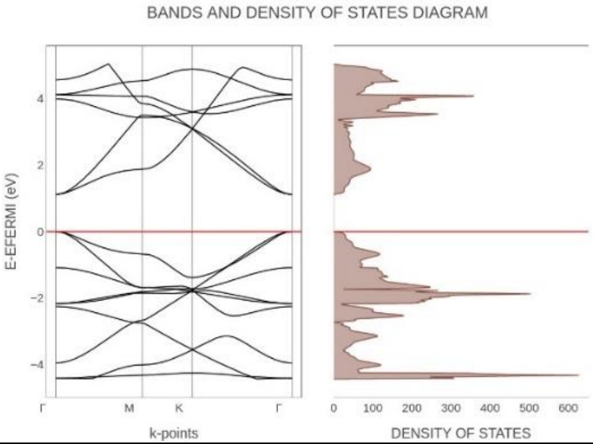
BANDS AND DENSITY OF STATES DIAGRAM
E-EFERMI (eV)
k-points
DENSITY OF STATES

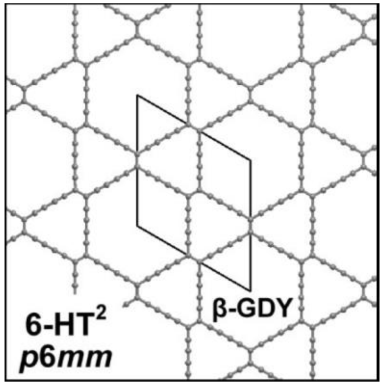
β-GDY
6-HT²
p6mm

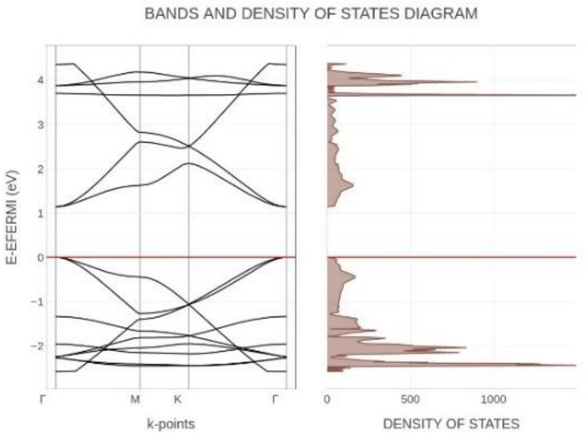
BANDS AND DENSITY OF STATES DIAGRAM
E-EFERMI (eV)
Γ M K Γ
k-points
DENSITY OF STATES

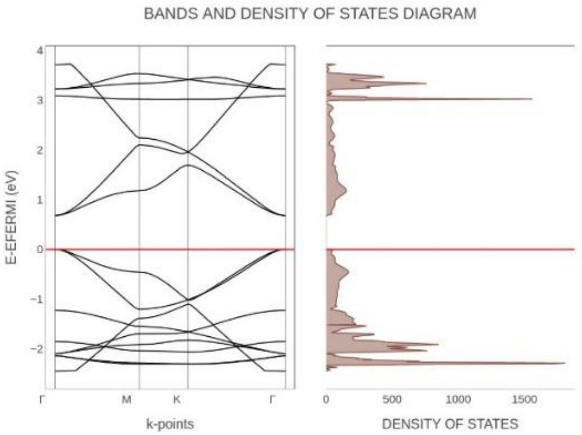
BANDS AND DENSITY OF STATES DIAGRAM
E-EFERMI (eV)
Γ M K Γ
k-points
DENSITY OF STATES

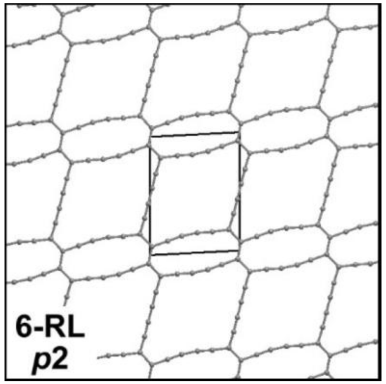
6-RL
p2

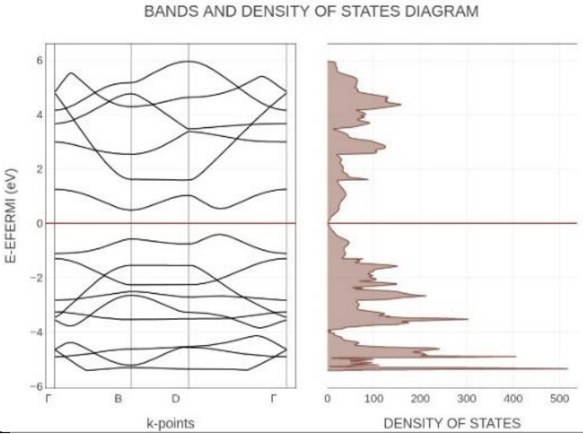
BANDS AND DENSITY OF STATES DIAGRAM
E-EFERMI (eV)
Γ B D Γ
k-points
DENSITY OF STATES

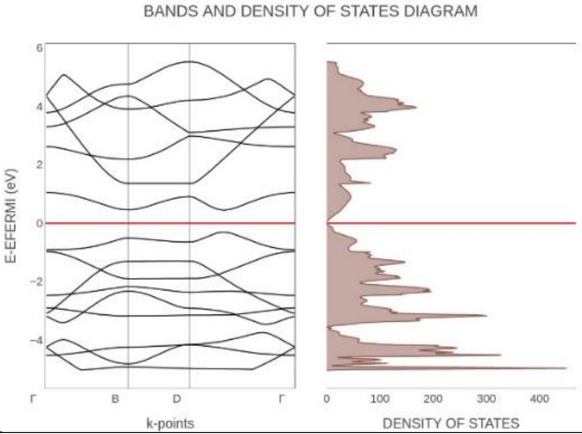
BANDS AND DENSITY OF STATES DIAGRAM
E-EFERMI (eV)
Γ B D Γ
k-points
DENSITY OF STATES

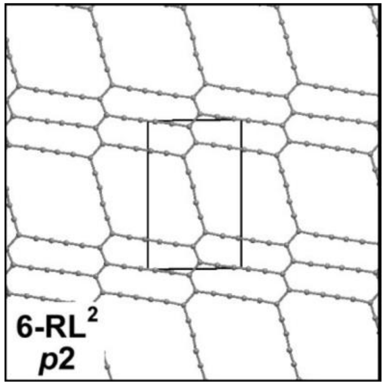
6-RL²
p2

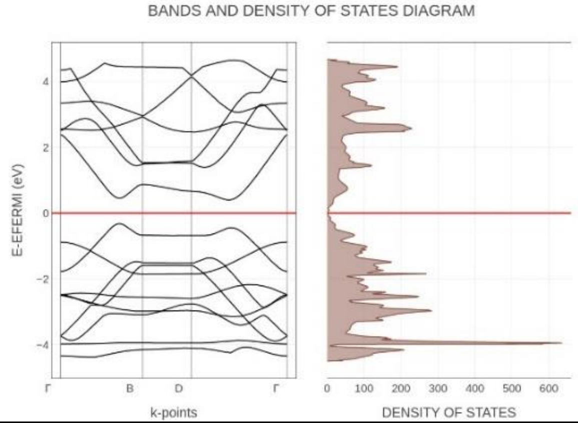
BANDS AND DENSITY OF STATES DIAGRAM
E-EFERMI (eV)
Γ B D Γ
k-points
DENSITY OF STATES

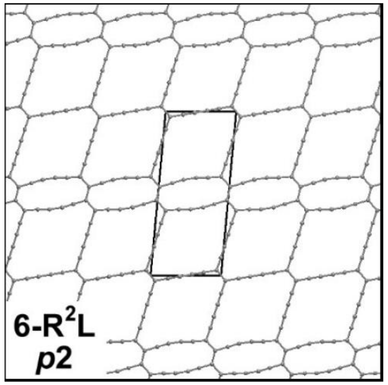 6-R^{2}L *p*2	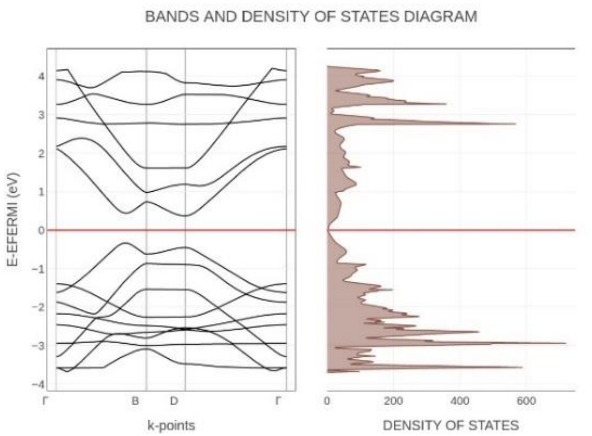 BANDS AND DENSITY OF STATES DIAGRAM E-EFERMI (eV) k-points DENSITY OF STATES	
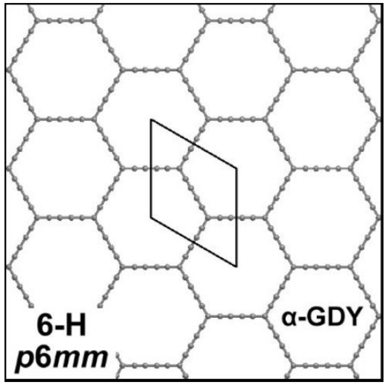 6-H *p6mm* α-GDY	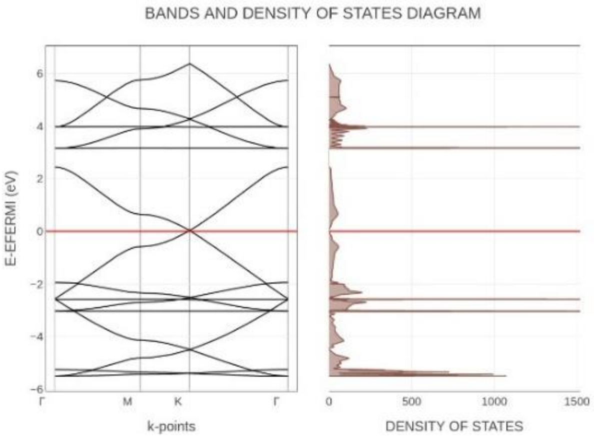 BANDS AND DENSITY OF STATES DIAGRAM E-EFERMI (eV) k-points DENSITY OF STATES	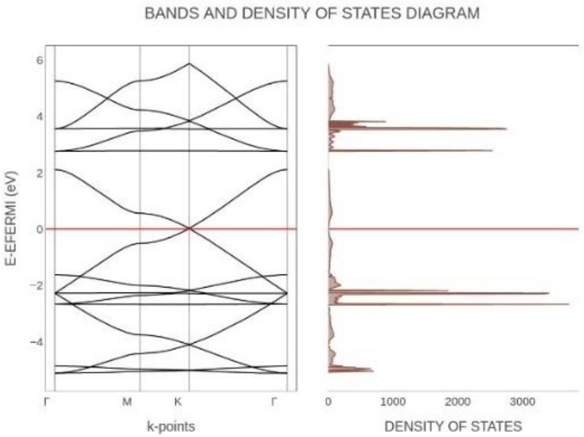 BANDS AND DENSITY OF STATES DIAGRAM E-EFERMI (eV) k-points DENSITY OF STATES

# in Tab 1	in red the new ones	Rel Energy kcal/mol	Area (Graphene 5.26Å^2)	density atomi/angs^2 graphene 0.380	SPGR	Plane group	C(sp2)m X(sp)n	ratio sp/sp2	Z	Pearson Symbol	# of C sp2 in the primitive cell	RINGS in the primitive cell	Other names	JETP 2015 (ref. 16) JPCC 2019 grazynes (ref. 56)	npj Comp Mat 2018 (ref. 17)	Phys. Status Solidi RRL 2020 (ref. 18)	J Chem Phys 1987 (ref.15)
1	6-h^{3}L	14,15	34,5	0,348	Pmmm	p2mm	C X2	0,5	4	oP12	8	[6]$_3$[14]		[3],[2]-grazyne = 4-h^{3}L			
2	6-h^{2}L	16,81	58,2	0,344	Cmmm	c2mm	C2 X3	0,66	4	oS20	6	[6]$_2$[14]		[2],[2]-grazyne = 4-h^{2}L			
3	6-h^{2}L^2	20,55	48,6	0,329	Pmma	p2mg	C X	1	8	oP16	8	[6]$_2$[14]$_2$					
4	6-hL	20,59	24,3	0,330	Pmmm	p2mm	C X	1	4	oP8	4	[6][14]		[1],[2]-grazyne = 4-hL			
5	6-hT^2	21,13	77,1	0,233	P6/mmm	p6mm	C2 X	2	6	hP18	6	[6][18]$_2$	γ-GDY graphdyne	γ1-graphyne-2	6-hT^2	191-E6Y12-1	6,6,6- = 4-hT^2 6,6,6-GDY
6	6-hRL^2	22,65	191,9	0,250	Cmmm	c2mm	C2 X	2	16	oS48	8	[6][14]$_2$[22]					
7	6-hT^{2}R	22,69	131,1	0,214	Pmmm	p2mm	C5 X2	2,5	4	oP28	8	[6][18]$_2$[22]		β2-γ2-graphyne = 4-hT^{2}R	6-hT^{2}R	47-E8Y20-0	6,6,12- = 4-hT^{2}R 6,6,18-GDY
8	6-hL^2	23,11	87,3	0,321	Cmmm	c2mm	C4 X3	1,33	4	oS28	6	[6][14]$_2$			>25Å		
9	6-hL^3	24,06	63,1	0,317	Pmmm	p2mm	C3 X2	1,5	4	oP20	8	[6][14]$_3$					
10	6-hR^3	24,07	166,5	0,192	P6/mmm	p6mm	C3 X	3	8	hP32	8	[6][22]$_3$			6-hR^3	191-E12Y12-1	
11	6-RL^2	24,73	87,2	0,252	P2/m	p2	C8 X3	2,66	2	mP22	6	[14]$_2$[22]					
12	6-RL	24,73	72,7	0,220	P2/m	p2	C3 X	3	4	mP16	4	[14][22]					
13	6-R^{2}L	25,03	127,2	0,204	P2/m	p2	C10 X3	3,33	2	mP26	6	[14][22]$_2$					
14	6-HT^{2}L	25,07	202,4	0,178	Pmmm	p2mm	C7 X2	3,5	4	oP36	8	[14][18]$_2$[30]	δ-GDY	β1-γ2-graphyne = 4-HT^{2}L	[4-HT^{2}L]		
15	6-HT^2	25,4	184,6	0,163	P6/mmm	p6mm	C4 X	4	6	hP30	6	[18]$_2$[30]	β-GDY	β1-graphyne-2	6-HT^2	191-E6Y24-1	12,12,12- = 4-HT^2 18,18,18-GDY
16	6-HL^3	25,41	165,6	0,193	P6/mmm	p6mm	C3 X	3	8	hP32	8	[14]$_3$[30]			[4-HL^3]	191-E8Y24-1	
17	6-R	25,48	109,4	0,183	Cmmm	c2mm	C4 X	4	4	oS20	2	[22]	carbon ene-yne CEY	β2-graphyne-2	6-R	65-E4Y16-0	14,14,14- = 4-R 22,22,22-GDY
18	6-HR^{2}L	25,85	474,9	0,168	Cmmm	c2mm	C4 X	4	16	oS80	8	[14][22]$_2$[30]			>25Å		
19	6-HR^3	26,11	276,1	0,159	Pmmm	p2mm	C9 X2	4,5	4	oP44	8	[22]$_3$[30]			>25Å		
20	6-L	26,18	38,9	0,309	Cmmm	c2mm	C2 X	2	4	oS12	2	[14]		γ2-graphyne-2		65-E4Y8-0 γ2-graphdiyne	
21	6-HR^2	26,3	442,5	0,154	Cmmm	c2mm	C14 X3	4,66	4	oS68	6	[22]2[30]		α-β2-graphyne Fig 4b = 4-HR^2	>25Å		
22	6-HR	26,63	166,7	0,144	Pmmm	p2mm	C5 X	5	4	oP24	4	[22][30]		α-β2-graphyne Fig 4a = 4-HR	6-HR	47-E4Y20-0	14,14,18- = 4-HR 22,22,30-GDY
23	6-H^{2}R^2	26,63	333,5	0,144	Pmma	p2mg	C5 X	5	8	oP48	8	[22]$_2$[30]$_2$			>25Å		
24	6-H^{2}R	26,92	558,2	0,136	Cmmm	c2mm	C16 X3	5,33	4	oS76	6	[22][30]$_2$			>25Å		
25	6-H^{3}R	27,05	391,5	0,133	Pmmm	p2mm	C11 X2	5,5	4	oP52	8	[22][30]$_3$			>25Å		
26	6-H	27,39	112,5	0,124	P6/mmm	p6mm	C6 X	6	2	hP14	2	[30]	α-GDY	α-graphyne-2	6-H	191-E2Y22-0	18,18,18- = 4-H 30,30,30-GDY

Table S2: Crystallographic informations for all the 26 2D GDY-based crystals investigated in the work
